# Periampullary Variceal Bleeding: An Atypical Complication of Portal Hypertension

**DOI:** 10.1155/2018/4643695

**Published:** 2018-05-03

**Authors:** Zahava C. Farkas, Priyanka Chugh, Shalom Frager, Khwaja F. Haq, Muhammad Ali Khan, Shantanu Solanki, Edward Esses, Gregory Veillette, Roxana Bodin

**Affiliations:** ^1^Department of Medicine, New York Medical College at Westchester Medical Center, Valhalla, NY, USA; ^2^Division of Gastroenterology and Hepatobiliary Disease, New York Medical College at Westchester Medical Center, Valhalla, NY, USA; ^3^Division of Gastroenterology and Hepatobiliary Disease, University of Tennessee Health Science Center, Memphis, TN, USA; ^4^Internal Medicine, New York Medical College at Westchester Medical Center, Valhalla, NY, USA; ^5^Department of Radiology, New York Medical College at Westchester Medical Center, Valhalla, NY, USA; ^6^Department of Hepatobiliary Surgery, New York Medical College at Westchester Medical Center, Valhalla, NY, USA

## Abstract

Variceal bleeding remains a fatal complication of portal hypertension. Periampullary varices are rare and, due to their location, are difficult to diagnose and treat. Similar to esophagogastric varices, they are the result of high portosystemic pressures secondary to intrahepatic causes such as cirrhosis and extrahepatic causes such as portal or splenic vein thrombosis. We report a case of a periampullary varix resulting in hemobilia during endoscopic retrograde cholangiopancreatography (ERCP).

## 1. Introduction

A periampullary varix, or dilated venous collateral surrounding the ampulla of vater, is a rare complication of portal hypertension. In our review of the available literature, only 1–3% of variceal bleeding in cirrhotic patients is attributed to duodenal varices with the most common sites being the duodenal bulb followed by the second part of the duodenum [1]. Although the vast majority of gastrointestinal (GI) bleeding occurs from ruptured esophagogastric varices, the portal enteropathy known to occur with portal hypertension makes the small bowel, including the region of the duodenal papilla, a potential source of bleeding.

This rare form of ectopic varix should be considered in patients with portal hypertension who are undergoing an ERCP and in those with upper GI bleeding without obvious stigmata of portal hypertension and a normal upper GI endoscopy. In some patients this may be the first manifestation of underlying portal hypertension, and therefore, maintaining a high clinical suspicion may reduce mortality rates and improve patient outcomes. The following case is presented to emphasize the clinical challenges of a periampullary varix resulting in GI bleeding during an ERCP.

## 2. Case Report

A 37-year-old woman with chronic portal vein thrombosis and cavernous transformation presented due to a recurrent episode of hematemesis. She had previously undergone multiple esophageal band ligations for esophageal varices seen on esophagogastroduodenoscopy (EGD). Ultrasonography showed elevated portal pressures and magnetic resonance cholangiopancreatography (MRCP) showed portal vein thrombosis. In addition, there were dilated periportal varices (cavernous transformation) exerting mass effect on the extrahepatic common bile duct and severe intrahepatic biliary ductal dilation involving all hepatic segments. Liver enzymes were persistently elevated in a cholestatic pattern: aspartate aminotransferase (AST) 64 U/L, alanine aminotransferase (ALT) 48 U/L, alkaline phosphatase (ALK) 303 U/L, total bilirubin (Tbili) 8.4 mg/dl, albumin 3.8 g/dl, and prothrombin time 11.9 seconds.

The liver was noncirrhotic on biopsy. Computed tomography (CT) of the abdomen showed large varices in the porta hepatis and portal vein thrombosis that appeared chronic in nature with cavernous transformation. Operative management with a mesocaval shunt was planned with the hopes of reducing portal pressure and preventing recurrent GI bleeding, portal biliopathy, and secondary cirrhosis. A selective shunt, such as a splenorenal shunt, selects nonintestinal flow to be shunted to the systemic venous drainage and has the advantage of reducing the incidence of encephalopathy. This type of shunt, however, would not have adequately reduced variceal pressures and therefore the decision was made to proceed with a mesocaval shunt.

**Figure 1:**
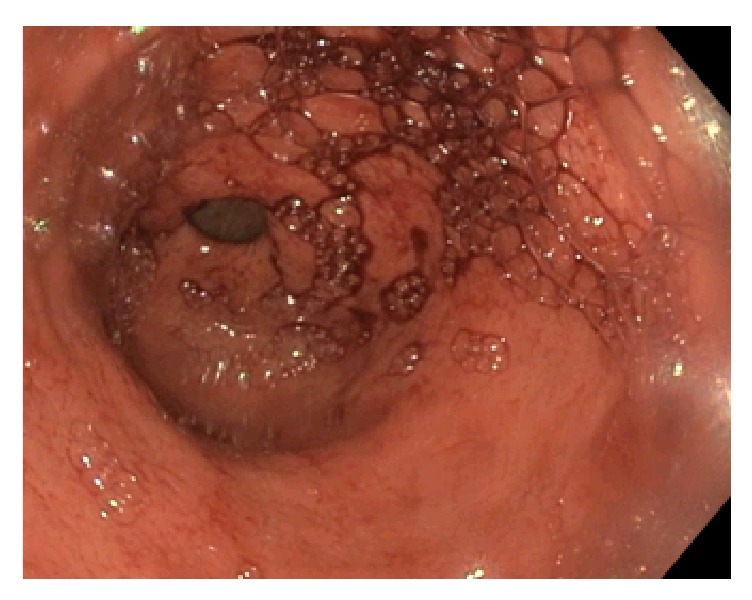
Hemobilia from a ruptured periampullary varix seen during an endoscopic retrograde cholangiopancreatography (ERCP).

**Figure 2:**
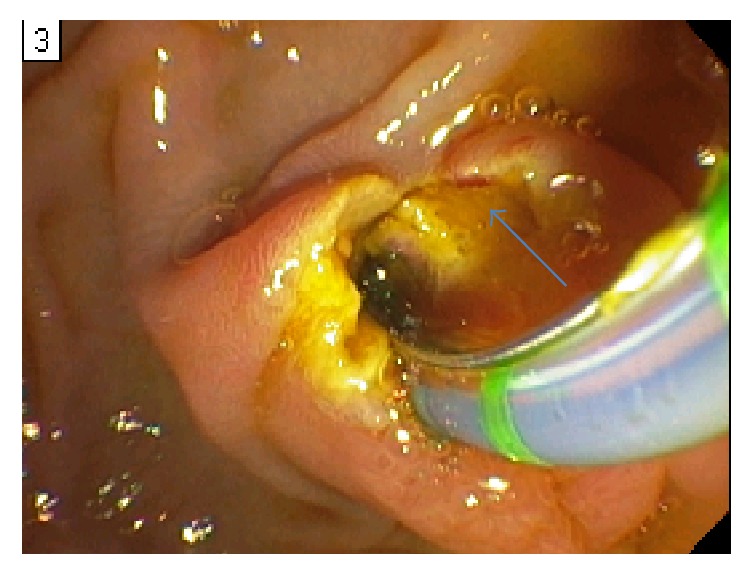
A duodenal varix (blue arrow) seen in the periampullary region on retroflexion during endoscopic retrograde cholangiopancreatography (ERCP).

**Figure 3:**
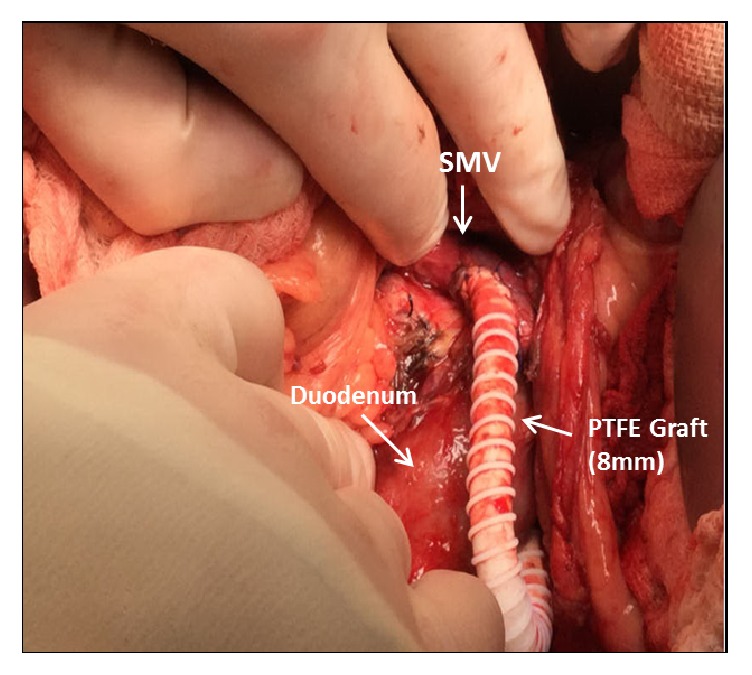
Intraoperative photo demonstrating the mesocaval bypass. An 8 mm ringed polytetrafluoroethylene (PTFE) graft was used to shunt blood flow from the proximal superior mesenteric vein (SMV) to the infrarenal IVC.

**Figure 4:**
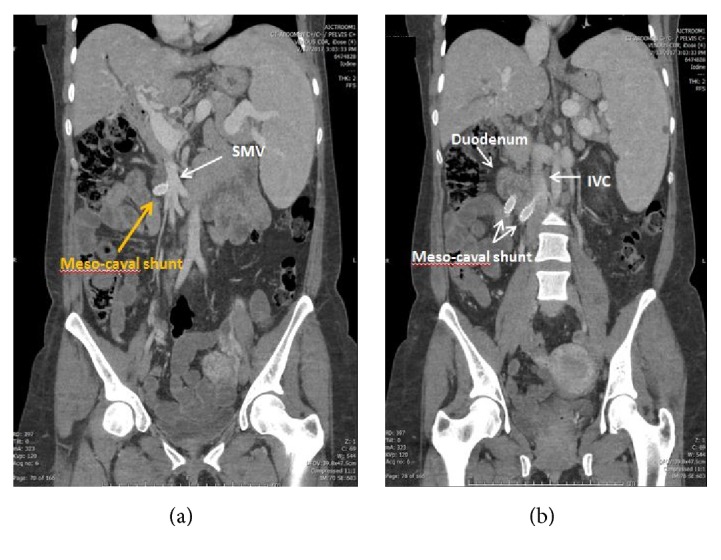
Coronal view on computed tomography showing the mesocaval shunt (yellow arrow) originating from the SMV and inserting into the infrarenal IVC.

The patient underwent an ERCP to better assess the biliary anatomy, as part of the preoperative evaluation for a shunt. During the procedure, she developed active hemobilia (Figure 1) from a newly discovered periampullary varix (Figure 2). A biliary covered metal stent was immediately placed, but without hemostasis. Due to ongoing bleeding that required activation of a massive transfusion protocol, an exploratory laparotomy was performed with emergent mesocaval shunt placement (Figures 3 and 4). An 8 mm ringed polytetrafluoroethylene (PTFE) graft was used to create a mesocaval shunt between the proximal superior mesenteric vein (SMV) and the infrarenal inferior vena cava (IVC). Portal pressures decreased by 8 mmHg after shunting and the bleeding stopped. She remained hemodynamically stable and was discharged ten days postoperatively. Follow-up mesocaval shuntogram showed a patent shunt with an outflow pressure of 13 mm Hg at the IVC and a portosystemic pressure gradient of 12 mm Hg. Two months postoperatively, she had an AST of 56 U/L, ALT 51 U/L, ALK 416 U/L, and Tbili 0.9 mg/dl. To date, she has had no episodes of hepatic encephalopathy.

## 3. Discussion

The second portion of the duodenum contains the major duodenal papilla and receives drainage from the pancreatic and common bile ducts through the ampulla of vater. Venous blood return from this area is through the pancreaticoduodenal vein to the gastroduodenal vein and ultimately into portal circulation through the splenic or superior mesenteric vein [2]. Elevated portal pressures can result in dilated tortuous vessels surrounding the ampulla, known as a periampullary varix. Similar to esophagogastric varices, they are the result of high portosystemic pressures secondary to intrahepatic causes such as cirrhosis and extrahepatic causes such as portal or splenic vein thrombosis.

The above reported case is an example of portal hypertensive biliopathy, defined as changes in the biliary tract in patients with portal hypertension due to extrahepatic portal vein obstruction [3]. Esophagogastric varices are the most common complication of portal hypertension; however, portosystemic venous collaterals can occur anywhere in the GI tract and are known as ectopic varices. These varices are less common and account for between 1% and 5% of all variceal bleeding. Although bleeding from ectopic varices is rare, it is generally massive and life-threatening [4].

Duodenal varices, such as the periampullary varix seen in this case, are most commonly found in the duodenal bulb and second portion of the duodenum. They are especially clinically relevant in patients known to have elevated portal pressures who are undergoing endoscopic intervention such as an ERCP. Appropriate preprocedure planning, using a multidisciplinary team approach, is important as mortality rates from bleeding duodenal varices are reportedly as high as 40% [5, 6].

Endoscopic variceal ligation (EVL) and endoscopic injection sclerotherapy (EIS) are the mainstay of treatment for bleeding esophageal varices; however, there are no widely accepted guidelines for treatment of a duodenal varix [6]. Isolated case reports describe treatment of bleeding duodenal varices using band ligation, injection sclerotherapy, decompressive procedures such as a mesocaval shunt, and surgical procedures like oversewing the varices [7, 8]. Interventional radiology treatment options for duodenal varices include transjugular intrahepatic portosystemic shunt (TIPS), balloon-occluded retrograde transvenous obliteration (BRTO), and percutaneous transhepatic obliteration (PTO). In the above reported case, a mesocaval shunt was performed to decrease pressures and control the bleeding. A mesocaval shunt reduces portal pressures by forming an anastomosis between the superior mesenteric vein and the inferior vena cava, thereby diverting flow away from the portal system.

In conclusion, this case highlights the importance of recognizing more rare complications of portal hypertension such as a periampullary varix. Appropriate foresight into such complications is necessary as bleeding from ectopic varices is often massive and achieving hemostasis is difficult. A multidisciplinary team including a skilled endoscopists, a hepatobiliary surgeon, and an interventional radiologist should be involved early on, in anticipation of these potential complications.
